# Addition of the Aβ42/40 ratio to the cerebrospinal fluid biomarker profile increases the predictive value for underlying Alzheimer’s disease dementia in mild cognitive impairment

**DOI:** 10.1186/s13195-018-0362-2

**Published:** 2018-03-20

**Authors:** Inês Baldeiras, Isabel Santana, Maria João Leitão, Helena Gens, Rui Pascoal, Miguel Tábuas-Pereira, José Beato-Coelho, Diana Duro, Maria Rosário Almeida, Catarina Resende Oliveira

**Affiliations:** 1 Laboratory of Neurochemistry, Neurology Department, Centro Hospitalar e Universitário de Coimbra, 3000-075 Coimbra, Portugal; 2 Center for Neuroscience and Cell Biology, University of Coimbra, 3004-504 Coimbra, Portugal; 3 Faculty of Medicine, University of Coimbra, 3000-548 Coimbra, Portugal; 4 Dementia Clinic, Neurology Department, Centro Hospitalar e Universitário de Coimbra, 3000-075 Coimbra, Portugal; 5 Research & Development Unit, Centro Hospitalar e Universitário de Coimbra, 3000-075 Coimbra, Portugal

**Keywords:** Mild cognitive impairment, CSF biomarkers, Aβ42/40 ratio, Alzheimer’s disease

## Abstract

**Background:**

Cerebrospinal fluid (CSF) biomarkers have been used to increase the evidence of underlying Alzheimer’s disease (AD) pathology in mild cognitive impairment (MCI). However, CSF biomarker-based classification often results in conflicting profiles with controversial prognostic value. Normalization of the CSF Aβ42 concentration to the level of total amyloid beta (Aβ), using the Aβ42/40 ratio, has been shown to improve the distinction between AD and non-AD dementia. Therefore, we evaluated whether the Aβ42/40 ratio would improve MCI categorization and more accurately predict progression to AD.

**Methods:**

Our baseline population consisted of 197 MCI patients, of which 144 had a follow-up ≥ 2 years, and comprised the longitudinal study group. To establish our own CSF Aβ42/40 ratio reference value, a group of 168 AD-dementia patients and 66 neurological controls was also included. CSF biomarker-based classification was operationalized according to the framework of the National Institute of Aging–Alzheimer Association criteria for MCI.

**Results:**

When using the core CSF biomarkers (Aβ42, total Tau and phosphorylated Tau), 30% of the patients fell into the high-AD-likelihood (HL) group (both amyloid and neurodegeneration markers positive), 30% into the low-AD-likelihood group (all biomarkers negative), 28% into the suspected non-Alzheimer pathophysiology (SNAP) group (only neurodegeneration markers positive) and 12% into the isolated amyloid pathology group (only amyloid-positive). Replacing Aβ42 by the Aβ42/40 ratio resulted in a significant increase in the percentage of patients with amyloidosis (42–59%) and in the proportion of interpretable biological profiles (61–75%), due to a reduction by half in the number of SNAP cases and an increase in the proportion of the HL subgroup. Survival analysis showed that risk of progression to AD was highest in the HL group, and increased when the Aβ42/40 ratio, instead of Aβ42, combined with total Tau and phosphorylated Tau was used for biomarker-based categorization.

**Conclusions:**

Our results confirm the usefulness of the CSF Aβ42/40 ratio in the interpretation of CSF biomarker profiles in MCI patients, by increasing the proportion of conclusive profiles and enhancing their predictive value for underlying AD.

**Electronic supplementary material:**

The online version of this article (10.1186/s13195-018-0362-2) contains supplementary material, which is available to authorized users.

## Background

Alzheimer’s disease (AD) is the leading cause of dementia worldwide and the most common neurodegenerative disease, affecting 4.6–8.7% of people over the age of 60 [1]. The pathophysiological process of AD is thought to begin many years before its clinical diagnosis [2, 3] and is generally hypothesized to be initiated by abnormal amyloid processing, followed by neuronal dysfunction and structural brain changes, which ultimately lead to cognitive impairment and dementia [4]. The interest in capturing the earliest stages of AD has been supported by the development of biomarkers of the disease like the core cerebrospinal fluid (CSF) AD biomarkers, amyloid positron emission tomography (PET) imaging and evidence of hippocampal atrophy on MRI. These biomarkers, reflecting both amyloid deposition and neuronal injury, have been incorporated into new diagnostic criteria, like those proposed by the National Institute of Aging–Alzheimer Association (NIA-AA) for AD dementia [5], mild cognitive impairment (MCI) [6] or preclinical states [7].

Core CSF biomarkers for AD are Aβ42, which is found in low concentrations in AD, probably reflecting brain amyloid deposition, total tau (t-Tau) at high concentrations representing cortical neuronal loss and phosphorylated tau (p-Tau) also at high concentrations, reflecting cortical tangle formation [8]. These markers have shown high diagnostic accuracy for established AD [9], and they may also be used to identify AD before onset of dementia at the stage of MCI, as shown in both single-center studies [10, 11] and large-scale heterogeneous multicenter studies [12–14]. However, low specificity regarding the distinction between AD and other types of degenerative dementias is still an issue and concern remains regarding the inter-laboratory variability of these CSF biomarkers and the lack of harmonization between centers [15]. In fact, several international standardization initiatives have already been launched to address these standardization issues [16, 17], and major advancements have been made in the field [18].

Recently, in order to improve the accuracy for AD diagnosis, other CSF biomarkers related to amyloid-beta (Aβ) metabolism have been studied [19]. Aβ is produced by the sequential proteolytic cleavage of amyloid precursor protein (APP) by β-secretases and γ-secretases [20], resulting in at least five different C-terminally truncated Aβ isoforms. The most abundant Aβ isoform in CSF is Aβ40 [19] which is less prone to aggregation, and thus, at least theoretically, a more direct measure of total brain Aβ content. It is thus conceivable that Aβ42 concentration, the Aβ isoform with a higher aggregation tendency, depends not only on the physiologic status (presence or absence of amyloid aggregates) but also on the total amount of Aβ peptides in the CSF, reflecting different efficiency of APP processing. Therefore, the use of the Aβ42/40 ratio is thought to more accurately reflect the changes in Aβ metabolism in AD than Aβ42 alone, as it corrects for individual baseline differences in both high and low amyloid-producing individuals [21]. Indeed, results from several groups demonstrated that normalization of the CSF Aβ42 concentration to the level of total Aβ peptides, using the Aβ42/40 ratio, improved the distinction between dementia and controls [22, 23] and also between AD and non-AD dementia [24–26], particularly in cases with ambiguous core CSF biomarker profiles (i.e., isolated reduction of Aβ42 or elevation of t-Tau/p-Tau) [27–29]. Recently, the CSF Aβ42/40 ratio has also been shown to be superior to Aβ42 alone in reflecting amyloid PET status (positive vs negative) [26, 30]. The added value of the CSF Aβ42/40 ratio for predicting AD in patients with MCI has so far been less studied. Hansson et al. [31] followed 131 MCI patients for 4–6 years and showed that the Aβ42/40 ratio was better than the Aβ42 concentration in identifying incipient AD in MCI. On the contrary, Parnetti et al. [32], in a study employing 90 MCI patients followed for up to 4 years, stated that the performance of the Aβ42/40 ratio was not superior to Aβ42 alone and that the Aβ42/p-tau ratio was the best parameter to predict conversion to AD in MCI patients. Also, in a smaller study, Brys et al. [33] had already shown that the Aβ42/40 ratio was inferior to p-Tau alone in predicting decline from MCI to AD. Very recently, a multicenter memory clinic cohort study from the German Dementia Competence Network [34], with 115 MCI patients, also indicated that Aβ42/40 ratio was not consistently superior to Aβ42 alone for predicting short-time progression to AD.

The NIA-AA guidelines for MCI due to AD propose categorizing MCI according to the individual likelihood of underlying AD pathophysiology, according to their biomarker profile [6]. In these guidelines, the highest likelihood category is characterized by biomarker findings pointing to the presence of AD pathophysiology, whereas the lowest likelihood category is characterized by findings not typical for AD. This categorization also includes subgroups of conflicting biomarker results, namely patients with biomarkers positive for amyloidosis but negative for neurodegeneration and patients with normal amyloid markers but positive for neurodegeneration. A number of studies, using CSF Aβ42, t-Tau and p-Tau, as well as imaging markers, have investigated the prognostic relevance of these biomarker-based categories in MCI patients [35–42]. General agreement exists as to the risk of progression to AD being higher in patients with all biomarkers positive for AD and lowest in patients with no positive biomarkers for AD. However, the biological significance and the prognosis of patients who fall into conflicting biomarker categories are still controversial.

Considering the data described, showing the relevance of Aβ40 inclusion in the panel of CSF biomarkers as a way of reducing diagnostic uncertainty, we hypothesize that using the Aβ42/40 ratio would significantly improve MCI categorization according to the NIA-AA guidelines by reducing the number of patients with conflicting biomarker results. Therefore, in this study, we propose to: first, using a group of AD patients and neurological controls, establish a cutoff value for the CSF Aβ42/40 ratio in our population; second, in an amnestic MCI cohort, assess the changes in MCI patients’ NIA-AA criteria classification induced by the inclusion of the Aβ42/40 ratio in their CSF biomarker profile; and, third, evaluate the prognostic value of this new classification for AD-type dementia at follow-up.

## Methods

### Subjects

This study included 431 subjects (168 AD-dementia patients, 197 MCI patients and 66 neurological controls) for whom CSF-AD biomarker assessment, including Aβ40, was available.

AD-dementia and MCI patients were recruited at the Dementia Clinic, Neurology Department of Coimbra University Hospital, Coimbra, Portugal. The baseline study and follow-up protocol have already been published elsewhere [43]. Patients were enrolled in a systematic way and had biannual clinical observation and annual neuropsychological and functional evaluations. All patients underwent a thorough biochemical, neurological and imaging (CT or MRI and SPECT) evaluation. PET and genetic studies were more restricted, although considered in younger patients. At baseline, a neurologist completed a medical history with the patient and the caregiver, and conducted a general physical, neurological and psychiatric examination as well as a comprehensive diagnostic battery protocol, including: cognitive instruments such as the Mini Mental State Examination (MMSE) [44] Portuguese version [45], The Montreal Cognitive Assessment (MoCA) [46] Portuguese version [47], the Alzheimer Disease Assessment Scale—Cognitive (ADAS-Cog) [48, 49] Portuguese version [50] and a comprehensive neuropsychological battery with normative data for the Portuguese population (Lisbon Battery for Dementia Assessment (BLAD)) [51] exploring memory (Wechsler Memory Scale subtests) and other cognitive domains (including language, praxis, executive functions and visuoconstrutive tests); and standard staging scales which provide objective information about subject performance in various domains, including the Clinical Dementia Rating scale (CDR) [52] for global staging, the Disability Assessment for Dementia (DAD) [53, 54] for evaluation of functional status and the Neuropsychiatric Inventory (NPI) [55, 56] to characterize the psychopathological profile, including the presence of depression. All of the available information (baseline cognitive test, staging scales, clinical laboratory and imaging studies) was used to reach a consensus research diagnosis. A similar approach was used for follow-up annual evaluations.

MCI patients included in this study were of the amnestic type and the diagnosis was made in accordance with the criteria defined by Petersen et al. [57] and, more recently, the framework for MCI due to AD proposed by NIA-AA criteria [6]. Petersen et al.’s criteria were operationalized as follows: a subjective complaint of memory decline (reported by the subject or an informant); an objective memory impairment (considered when scores on standard Wechsler memory tests were > 1.5 SDs below age/education-adjusted norms) with or without deficits in other cognitive domains; normal general cognition suggested by normal scores for the MMSE and MoCA using the Portuguese cutoff scores [45, 58]; largely normal daily life activities, evaluated with a functional scale (DAD); and absence of dementia, indicated by a CDR rating of 0.5. All patients were in a stable condition, without acute comorbidities. As exclusion criteria for enrolment, we considered a significant underlying medical or neurological illness revealed by laboratory tests or imaging; a relevant psychiatric disease, including major depression, suggested in the medical interview and confirmed by the GDS; and CT or MRI demonstration of significant vascular burden [59] (large cortico-subcortical infarct; extensive subcortical white matter lesions superior to 25%; unilateral or bilateral thalamic lacunes; lacune in head of caudate nucleus; more than two lacunes).

MCI cases were followed up with this comprehensive protocol until they developed dementia, or until they had been cognitively stable for at least 2 years, comprise the longitudinal study group. This group was further dichotomized into those who were cognitively stable and those who developed dementia due to AD. Patients who developed types of dementia other than AD were further excluded from analysis. Conversion to AD required fulfilling clinical diagnostic criteria for probable AD (see later) and was confirmed by the coordinator of the clinical study. As these criteria are not fully operational and the conversion status decision has some uncertainty and subjectivity, patients in this study were classified as having undergone conversion based on: objective evidence, by cognitive testing, of decline to dementia using the MMSE, the MoCA and the ADAS-Cog scores and qualitative evaluation (i.e., impairment of memory plus another domain); and changes in global CDR rating from 0.5 to 1 or more, confirming the cognitive profile of dementia and loss of autonomy.

For the biomarker-based subject classification, we used the core CSF biomarkers for AD, operationalized according to the framework of the NIA-AA criteria for MCI and preclinical forms [6, 7, 60]. Subjects were classified into the low-AD-likelihood (LL) group if both amyloid (i.e., CSF Aβ42) and neuronal injury markers (i.e., CSF t-tau and p-tau) were normal, into the high-AD-likelihood (HL) group if both amyloid and at least one neuronal injury marker were abnormal, or into one of the two conflicting biomarker groups: the isolated amyloid pathology (IAP) group if the amyloid marker was abnormal and neuronal injury markers were normal, or the suspected non-Alzheimer pathophysiology (SNAP) group if at least one neuronal injury marker was abnormal and the amyloid marker was normal.

Dementia was diagnosed according to the *Diagnostic and Statistics Manual for Mental Disorders*—fourth edition text review (DSM-IV-TR) criteria [61], and AD according to the National Institute of Neurological and Communicative Disorders and Stroke–Alzheimer’s Disease and Related Disorders (NINCDS-ADRDA) criteria [62] and, more recently, the 2011 NIA-AA criteria [5]. These cases were classified as probable AD dementia according to clinical and neuroimaging features.

In this study we also included 66 neurological controls. Most of these individuals suffered from acute or chronic headaches, and a lumbar puncture was performed as part of their routine diagnostic evaluation in order to exclude bleeding or inflammation; in some cases, this procedure was considered in the investigation of a peripheral polyneuropathy. In both situations, the CSF cytochemical evaluation was normal and a major CNS disease was excluded. In their brief cognitive evaluation they showed no subjective cognitive complaints, were independent in their instrumental daily life activities and most of them were still professionally active.

### Laboratory determinations

CSF samples were collected from patients and neurological controls as part of their routine clinical diagnosis investigation. Preanalytical and analytical procedures were done in accordance with previously proposed protocols [63]. Briefly, CSF samples were collected in sterile polypropylene tubes, immediately centrifuged at 1800 × *g* for 10 min at 4 °C, aliquoted into polypropylene tubes and stored at –80 °C until analysis.

CSF Aβ42, t-Tau and p-Tau were measured separately, in duplicate, by commercially available sandwich ELISA kits (Innotest; Innogenetics/Fujirebio, Ghent, Belgium), as described previously [22, 64]. These assays were performed sequentially in a clinical routine setting between 2010 and 2017, with mean intra-assay coefficients of variation (CVs) of 4.2% for Aβ42, 4.5% for t-Tau and 4.2% for p-Tau and inter-assay CVs of 8.1% for Aβ42, 7.0% for t-Tau and 7.2% for p-Tau. CSF Aβ40 was also measured by ELISA, using the recently validated kit also from Fujirebio [65], following the manufacturer’s instructions. In our hands, the intra-assay CV of this method was 3.8 ± 1.8% (mean ± SD) and the inter-assay CV was 13.2 ± 4.0%, and therefore very similar to what has been reported [65]. Aβ40 assays were also performed in duplicate between November 2016 and March 2017.

External quality control of the assays was performed under the scope of the Alzheimer’s Association Quality Control Program for CSF Biomarkers [66]. In this study, we established cutoff values for core CSF-AD biomarkers for this particular population by employing receiver operating characteristics (ROC) curve analysis between AD-dementia patients and controls, as reported previously [64]. According to these cutoff values (Aβ42 = 585 pg/ml, t-Tau = 244 pg/ml, p-Tau = 38 pg/ml), core CSF-AD biomarkers were classified as normal/abnormal.

Blood samples were also collected from MCI and AD patients for Apolipoprotein E (APOE) genotyping. DNA was isolated from whole EDTA blood using a commercial kit (Roche Diagnostics GmbH, Manheim, Germany), as described by the manufacturer. The analysis of the two polymorphisms at codons 112 and 158 of the *APOE* gene (rs429358 and rs7412) was performed by PCR-RFLP assay, as described previously [67].

### Statistical analysis

Statistical analyses were performed using the Statistical Package for the Social Sciences (SPSS, version 20.0; IBM SPSS, Chicago, IL, USA). Normality of continuous variables was assessed by the Kolmogorov–Smirnov test. For normally distributed continuous variables, one-way ANOVA followed either by the Bonferroni post test (when variance was homogeneous between groups) or the Games–Howell post test (when variance was not homogeneous between groups) was performed to assess the statistical significance of the difference between means. When continuous variables did not show normal distribution, the Kruskal–Wallis test was used, followed by the Dunn–Bonferroni post-hoc test. For CSF biomarkers, age was entered as a covariate in the analysis. Group differences between categorical variables were examined using the χ^2^ test with Yate’s correction for small sample sizes (*n* < 30). McNemar’s test for paired proportions was used for testing differences between the proportions of cases with amyloidosis and with conclusive/ambiguous CSF biomarker profiles. ROC curve analysis was used to evaluate the diagnostic accuracy of the CSF markers or their ratios between AD-dementia patients and controls, and also of the predicted probabilities derived from the logistic regression models used to identify the best predictors of conversion to AD. The ROC curve was also used to establish the optimal cutoff value, by selecting the value that yielded the highest Youden index calculated as: sensitivity + (specificity – 1). The ROC curves were compared according to the AUC comparison method of Hanley and McNeil [68] using MedCalc (version 11.6; MedCalc Software, Mariakerke, Belgium). Binary logistic regression analysis (enter method) was used to identify predictive markers of conversion to AD, with conversion as the dependent variable and age, gender, education, follow-up time, APOE genotype, baseline MMSE, CSF Aβ42, Aβ40, t-Tau and p-Tau levels as independent variables. Variables with a regression coefficient significantly different from 0 (associated *p* < 0.05) were considered to be contributing significantly to the prediction of the outcome variable. Survival analysis was used to assess the probability of conversion to AD in the different MCI subgroups. Kaplan–Meier survival curves were plotted and the survival distributions of the different MCI subgroups were compared by the log-rank test. Survival time was calculated as the interval from the initial baseline evaluation to the diagnosis of dementia. For patients who remained nondemented, the survival time was censored at the date of the last clinical assessment. Cox proportional hazards models, corrected for age, gender, education, follow-up time, ApoE genotype and baseline MMSE score, were used to test the predictive ability for AD-type dementia of the different MCI groups.

## Results

### Characteristics of the study population

Demographic, clinical, genetic and biomarker data of the baseline study population are presented in Table 1. No differences in gender distribution were seen between groups, but the control group was significantly younger than the patient groups, and therefore age was entered as a covariate in the cognitive and CSF marker comparisons. Similar age of onset was seen between AD-dementia and MCI patients, but, as expected, AD-dementia patients presented with a more severe cognitive impairment (significantly lower MMSE and MoCA and higher ADAS-Cog scores) than MCI patients at baseline. The percentage of *APOE*-ε4 carriers in AD-dementia and MCI patients was over 40%, considerably higher than we showed previously in a Portuguese control population [69]. As reported previously [22, 64], CSF Aβ42 was lower and t-Tau and p-Tau were higher in AD-dementia patients compared to controls, while MCI patients had intermediate and significantly different values in relation to the two other groups. A significant increase in the t-Tau/Aβ42 ratio and a decrease in the Aβ42/p-Tau ratio in AD-dementia patients compared to controls was also observed, while MCI patients had intermediate and significantly different values from the other groups. CSF Aβ40 was similar between AD-dementia and controls, but was significantly higher in MCI patients in relation to AD-dementia patients only. This resulted in a decreased Aβ42/40 ratio in AD and MCI patients in relation to controls, and also in AD-dementia patients compared with the total MCI group.

**Table 1:** Demographic, clinical, genetic and biomarker data of the study population

	Controls (*n* = 66)	AD-dementia(*n* = 168)	MCI total(*n* = 197)	MCI-St(*n* = 74)	MCI-AD(*n* = 70)
Gender (M/F)	27/39	56/112	68/127	25/49	26/44
Age (years)	58.8 ± 11.9	68.1 ± 8.8***	67.1 ± 9.4***	65.2 ± 9.3*	71.0 ± 8.0***^,§§^
Age onset (years)		64.6 ± 8.7	64.2 ± 9.3	60.5 ± 9.4	68.8 ± 7.7^γ,§§§^
Education (years)		5.5 ± 4.0	6.0 ± 3.8	5.7 ± 3.6	5.8 ± 3.9
MMSE	28.4±1.8	17.2 ± 5.9***	25.9 ± 4.0***^,γγγ^	26.5 ± 4.9***^,γγγ^	25.1 ± 3.3***^,γγγ,§^
MoCA		10.5 ± 5.1	17.7 ± 5.6^γγγ^	19.9 ± 4.9^γγγ^	15.5 ± 5.3^γγ,§§§^
ADAS-Cog		26.0 ± 12.3	11.8 ± 6.1^γγγ^	9.0 ± 4.3^γγγ^	14.7 ± 6.3^γγγ,§§§^
*APOE*-ε4 (%)		46%	42%	28%	60%^§§§^
Aβ42 (pg/ml)	928 ± 435	455 ± 225***	689 ± 322***^,γγγ^	785 ± 309*^,γγγ^	476 ± 180***^,§§§^
Aβ40 (pg/ml)	10,055 ± 4314	9229 ± 3668	11,097 ± 4635^γγγ^	10,916 ± 4379^γγ^	10,157 ± 3381
Aβ42/40 ratio	0.100 ± 0.034	0.055 ± 0.029***	0.070 ± 0.039***^,γγγ^	0.083 ± 0.046*^,γγγ^	0.050 ± 0.020***^,§§§^
t-Tau (pg/ml)	196 ± 95	543 ± 363***	374 ± 269***^,γγγ^	253 ± 151^γγγ^	511 ± 307***^,§§§^
p-Tau (pg/ml)	33 ± 15	64 ± 37***	50 ± 28**^,γγγ^	38 ± 19^γγγ^	62 ± 30***^,§§§^
t-Tau/Aβ42	0.22 ± 0.11	1.53 ± 1.36***	0.74 ± 0.73***^,γγγ^	0.41 ± 0.39^γγγ^	1.18 ± 0.85***^,§§§^
Aβ42/p-Tau	32.0 ± 15.3	9.3 ± 8.2***	18.6 ± 14.2***^,γγγ^	25.7 ± 16.3^γγγ^	9.7 ± 6.6***^,§§§^

Data expressed as mean ± standard deviation, except for *APOE* expressed as percentage of ε4 carriers

MMSE and MoCA, higher scores correspond to better performance; ADAS-Cog, lower scores correspond to better performance. For MMSE and CSF biomarkers, data were adjusted for age

*AD* Alzheimer’s disease, *MCI* mild cognitive impairment, *MCI-St* stable mild cognitive impairment, *MCI-AD* mild cognitive impairment patients who progress to Alzheimer’s disease, *M* male, *F* female, *MMSE* Mini Mental State Examination, *MoCA* Montreal Cognitive Assessment, *ADAS-Cog* Alzheimer Disease Assessment Scale—Cognitive, *APOE* Apolipoprotein E, *Aβ42* 42-aminoacid isoform of amyloid beta, *Aβ40* 40-aminoacid isoform of amyloid beta, *t-Tau* total Tau protein, *p-Tau* hyperphosphorylated Tau protein

*p < 0.05 vs controls

**p < 0.01 vs controls

***p < 0.001 vs controls

^γ^*p* < 0.05 vs AD-dementia

^*γγ*^*p* < 0.01 vs AD-dementia

^*γγγ*^*p* < 0.001 vs AD-dementia

^§^*p* < 0.05 vs MCI-St

^§§^*p* < 0.01 vs MCI-St

^§§§^*p* < 0.001 vs MCI-St

### Establishment of a cutoff value for the CSF Aβ42/40 ratio

By comparing the Aβ42/40 ratio between AD-dementia patients and controls, as recommended by the STARD criteria [70], a cutoff value of 0.068 (with lower values indicative of AD) was established. This ratio had a sensitivity of 79% and a specificity of 86% to distinguish between AD-dementia patients and controls, with an AUC of 0.874 (95% CI 0.827–0.921). These accuracy parameters are similar to what we have reported previously for Aβ42 alone in a larger AD cohort [64] and are no different from that presented by Aβ42 alone in this particular AD-dementia population (sensitivity = 82%, specificity = 83%, AUC = 0.882, 95% CI 0.837–0.927, *p* = 0.748) (Additional file 1: Figure S1).

### Characterization of MCI biomarker-based subgroups according to the core CSF biomarkers

The MCI group was then classified into MCI subtypes according to their core CSF biomarkers (Aβ42, t-Tau and p-Tau): LL, 59 (29.9%); HL, 62 (31.5%); IAP, 21 (10.6%); and SNAP, 55 (27.9%). Noteworthy, the percentage of MCI patients with injury markers (59.3%) was higher than those with amyloidosis (42.1%). Table 2 presents the demographic, clinical, genetic and biomarker data of these subgroups. There were no significant differences regarding gender, years of education and time of follow-up, but the HL and SNAP patients were older at baseline and at onset of the symptoms (*p* = 0.001 vs LL group). Regarding the cognitive tests, both the MMSE and the MoCA mean scores were significantly lower in the HL group in comparison with the LL group (*p* < 0.001 and *p* = 0.004, respectively). Conversely, the ADAS-Cog mean score was higher both in the HL and SNAP groups vs the LL group (*p* = 0.005 and *p* = 0.015, respectively), again indicating greater cognitive impairment. As expected, differences were significant in terms of Aβ42 levels between HL and IAP vs SNAP and LL, as well as in terms of neuronal injury markers between SNAP and HL vs IAP and LL (*p* < 0.001 for all comparisons). Regarding Aβ40, a significant reduction was seen in the IAP group (*p* = 0.032 in relation to the HL group), while a significant increase was observed in the SNAP group in comparison with all the other groups (*p* < 0.001 for all comparisons). Interestingly, in cases with an ambiguous biological profile (IAP + SNAP), Aβ40 levels were significantly more dispersed (ranged between 2899 and 41,282 pg/ml) than in patients with conclusive core CSF biomarkers (LL + HL; ranged between 3516 and 28,908 pg/ml, *p* = 0.001). A significantly increased Aβ42/40 ratio was seen in the LL group (*p* < 0.001 for all comparisons), and also in the SNAP in comparison with the HL group (*p* < 0.001). Subjects in the HL group were also more often *APOE*-ε4 carriers (67%) than in all other groups (*p* < 0.001).

**Table 2:** Demographic, clinical, genetic and biomarker data of the MCI subgroups based on core CSF biomarkers

	Low-AD likelihood	High-AD likelihood	IAP	SNAP
*N* (%)	59 (29.9%)	62 (31.5%)	21 (10.6%)	55 (27.9%)
Gender (M/F)	17/42	26/36	8/13	17/38
Age (years)	62.7 ± 9.9	69.6 ± 7.7**	66.3 ± 9.7	69.4 ± 8.9**
Age onset (years)	59.0 ± 10.3	66.4 ± 7.8**	63.1 ± 8.1	67.4 ± 8.1**
Education (years)	6.5 ± 3.9	6.1 ± 4.1	5.1 ± 2.4	5.8 ± 4.1
MMSE	27.6 ± 2.6	24.3 ± 4.2***	25.1 ± 6.0	26.3 ± 3.2
MoCA	20.5 ± 5.0	16.4 ± 5.5**	16.3 ± 6.9	17.3 ± 4.9
ADAS-Cog	8.5 ± 4.6	12.7 ± 4.3**	13.3 ± 7.5	13.5 ± 7.5*
*APOE*-ε4 (%)	26%	64%***	24%^γγγ^	39%^γγ^
Aβ42 (pg/ml)	918 ± 243	405 ± 109***	403 ± 123***^,§§§^	867 ± 282^γγγ^
Aβ40 (pg/ml)	9608 ± 3219	10,945 ± 4191	8006 ± 3082^γ,§§§^	14,247 ± 5219***^,γγγ^
Aβ42/40 ratio	0.105 ± 0.040	0.040 ± 0.017***	0.066 ± 0.024***	0.061 ± 0.037***^,γγγ^
t-Tau (pg/ml)	169 ± 42	545 ± 274***	159 ± 67^γγγ,§§§^	488 ± 250***
p-Tau (pg/ml)	30 ± 9	68 ± 28***	28 ± 7^γγγ,§§§^	61 ± 27***
Follow-up time (years)	4.0 ± 3.3	4.0 ± 2.5	4.2 ± 4.1	3.6 ± 2.7

Data expressed as mean ± standard deviation, except for *APOE* expressed as percentage of ε4 carriers

*MCI* mild cognitive impairment, *AD* Alzheimer’s disease, *IAP* isolated amyloid pathology, *SNAP* suspected non-Alzheimer pathology, *M* male, *F* female, *MMSE* Mini Mental State Examination, *MoCA* Montreal Cognitive Assessment, *ADAS-Cog* Alzheimer Disease Assessment Scale—Cognitive, *APOE* Apolipoprotein E, *Aβ42* 42-aminoacid isoform of amyloid beta, *Aβ40* 40-aminoacid isoform of amyloid beta, *t-Tau* total Tau protein, *p-Tau* hyperphosphorylated Tau protein

MMSE and MoCA, higher scores correspond to better performance; ADAS-Cog, lower scores correspond to better performance

*p < 0.05 vs low-AD likelihood

**p < 0.005 vs low-AD likelihood

***p < 0.001 vs low-AD likelihood

^*γ*^*p* < 0.05 vs high-AD likelihood

^*γγ*^*p* < 0.01 vs high-AD likelihood

^γγγ^*p* < 0.001 vs high-AD likelihood

^*§§§*^*p* < 0.001 vs SNAP

### Effect of including the Aβ42/40 ratio in MCI biomarker-based classification

When we used the Aβ42/40 ratio, instead of Aβ42 alone, as a marker of amyloidosis, the MCI biomarker-based classification changed according to what is presented in Table 3. In MCI subtypes with already conclusive biomarker profiles (LL and HL), use of the Aβ42/40 ratio did not change their classification in 87% of cases (83% of LL and 90% of HL). Noteworthy, in 10/59 patients classified previously as LL, the Aβ42/40 ratio fell below the cutoff value and they were therefore classified as IAP; likewise, in 6/62 patients classified previously as HL, the Aβ42/40 ratio was within the normal range and changed their classification to SNAP. Regarding the subgroups with conflicting biomarker results, more pronounced changes were seen both in the IAP group (with 7/21 (33%) changing into the LL category) and particularly in the SNAP group (where 36/55 (66%) of patients had an abnormal Aβ42/40 ratio and therefore changed their classification to HL). Overall, addition of the Aβ42/40 ratio resulted in a redistribution of the MCI subtypes to 56 (28.4%) LL, 92 (46.7%) HL, 24 (12.2%) IAP and 25 (12.7%) SNAP (Table 3), therefore significantly improving the proportion of interpretable biological profiles from 61% to 75% (*p* = 0.001). Moreover, it significantly increased the percentage of MCI patients with amyloidosis from 42 to 59% (*p* < 0.001), therefore reaching a similar percentage to the patients with injury markers. Overall demographic, clinical and genetic differences between these new subgroups are similar to those presented in Table 2 (Additional file 2: Table S1). Interestingly, among patients classified as SNAP according to Aβ42 alone, those who changed their classification to HL according to the Aβ42/40 ratio had significantly lower Aβ42 than those who remained in the SNAP category (751 ± 148 *vs* 1088 ± 343 pg/ml, *p* < 0.001). A similar pattern was also seen in patients classified previously in the LL group who changed their classification to IAP in relation to those who remained in this category (Aβ42 = 758 ± 114 pg/ml vs Aβ42 = 951 ± 250 pg/ml, *p* = 0.015). Conversely, for patients previously classified into the HL group, significantly decreased t-Tau and p-Tau levels were found in those who changed their classification to SNAP (t-Tau = 310 ± 76 pg/ml, p-Tau = 42 ± 7 pg/ml) in relation to those who remained in this category (t-Tau = 570 ± 276 pg/ml, *p* = 0.001; p-Tau = 71 ± 28 pg/ml, *p* = 0.001). No other significant changes were observed in any of the demographic and clinical parameters between patients who changed their biomarker-based classification and those who did not.

**Table 3:** Distribution of Aβ42/40 ratio and further classification of MCI biomarker-based subgroups

	Aβ42/40 ratio < 0.068	Aβ42/40 ratio ≥ 0.068
LL (*n* = 59)	10 (17%)—IAP	49 (83%)—LL
IAP (*n* = 21)	14 (67%)—IAP	7 (33%)—LL
SNAP (*n* = 55)	36 (66%)—HL	19 (34%)—SNAP
HL (*n* = 62)	56 (90%)—HL	6 (10%)—SNAP

Data expressed as *n* (percentage) of patients

*Aβ42/40* 42-aminoacid isoform of amyloid beta/40-aminoacid isoform of amyloid beta, *LL* Low-AD likelihood, *IAP* isolated amyloid pathology, *SNAP* suspected non-Alzheimer pathology, *HL* high-AD likelihood, *AD* Alzheimer’s disease

### Longitudinal assessment of MCI patients

Of the 197 MCI patients enrolled, 36 had follow-up < 2 years, 13 were dropouts and four patients were excluded from the further analysis because, although their clinical presentation was amnestic MCI, they developed frontotemporal dementia. The remaining 144 subjects with follow-up ≥ 2 years (mean follow-up time 4.6 ± 2.9 years, range 2–15 years) comprise the longitudinal study group, which was further dichotomized into those who were cognitively stable in the last observation (74 (51%); MCI-St) and those who progressed to dementia due to AD (70 (49%); MCI-AD). Mean follow-up time was not different between patients who converted to AD (4.7 ± 3.3 years) and those who did not (4.5 ± 2.5 years, *p* = 0.990). Baseline demographic, clinical, genetic and biomarker characteristics of MCI-St and MCI-AD patients are presented in Table 1. No differences in gender distribution or years of education were seen between groups, but MCI-AD patients were older both at baseline observation and at disease onset than MCI-St patients. As expected, MCI-AD patients presented with a more severe cognitive impairment at baseline (significantly lower MMSE and MoCA and higher ADAS-Cog scores) than MCI-St patients, and were also more frequently *APOE*-ε4 carriers. Differences in baseline CSF Aβ42, t-Tau and p-Tau, and in Aβ42/40, t-Tau/Aβ42 and Aβ42/p-Tau ratios, but not in Aβ40, were seen between the two MCI groups. Aβ42, Aβ42/40 and Aβ42/p-Tau ratios were lower and t-Tau, p-Tau and the t-Tau/Aβ42 ratio were higher in MCI-AD patients, and were similar to the values presented by AD patients. MCI-St patients had comparable t-Tau, p-Tau, and t-Tau/Aβ42 and Aβ42/p-Tau ratios to the control group, while Aβ42 and the Aβ42/40 ratio were slightly but significantly lower than the controls. MCI-St patients also had significantly increased levels of Aβ40 in relation to AD patients.

### Conversion to AD in MCI biomarker-based subgroups

**Fig. 1:**
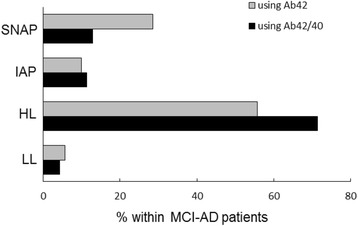
Comparison of MCI biomarker-based subgroups with clinical follow-up. Amongst mild cognitive impairment patients who progressed to Alzheimer’s disease during clinical follow-up (MCI-AD), the percentage of different biomarker-based subgroups was compared. Biomarker-based subgroups determined using either CSF Aβ42, t-Tau and p-Tau (gray bars) or Aβ42/40 ratio, t-Tau and p-Tau (black bars). LL low-AD likelihood, HL high-AD likelihood, IAP isolated amyloid pathology, SNAP suspected non-Alzheimer pathology; Aβ42 42-aminoacid isoform of amyloid beta, Aβ40 40-aminoacid isoform of amyloid beta

Conversion to AD in MCI categories based on their core CSF biomarkers (Aβ42, t-Tau and p-Tau) showed that subjects in the HL group were more prone to progress to AD (75.0%), than all other biomarker groups: SNAP, 55.6%; IAP, 43.8%; LL, 10.0% (*p* < 0.001) (Table 4). This profile did not change much when we used the new classification with the Aβ42/40 ratio alongside Tau and p-Tau: progression to AD during clinical follow-up occurred in 72% of HL patients and in only 8% of the LL group. Noteworthy, still approximately half of the patients within the IAP (44%) and SNAP (53%) subtypes progressed to AD during clinical follow-up (Table 4). Interestingly, in patients classified previously into the IAP subgroup, conversion to AD occurred in 58.3% (7 out of 12) of patients who remained in this subgroup, and in none of the patients who changed their classification to LL, after replacing Aβ42 by the Aβ42/40 ratio. This difference, however, failed to reach statistical significance (*p* = 0.146). In patients classified previously into the SNAP subgroup, conversion to AD occurred at a similar percentage both in the patients who remained classified as SNAP after Aβ42/40 ratio inclusion (5 out of 11, 45.5%) and those who changed their classification to HL (15 out of 25, 60.0%) (*p* = 0.656). Figure 1 shows the percentage of the different MCI biomarker-based subgroups, determined using either the core CSF biomarkers (Aβ42, t-Tau and p-Tau) or the Aβ42/40 ratio, t-Tau and p-Tau, amongst patients who progressed to AD during clinical follow-up. The percentage of LL and IAP subtypes within MCI-AD patients was low (6% and 11%, respectively) and was practically not influenced by the inclusion of Aβ42 (5.7% and 10.0%, respectively) or the Aβ42/40 ratio (4.3% and 11.4%, respectively) in their biomarker-based classification. On the contrary, the inclusion of the Aβ42/40 ratio significantly decreased the proportion of the SNAP subtype (from 28.6 to 12.9%) and increased the HL subtype (from 55.7 to 71.4%) within MCI-AD patients (*p* = 0.019).

**Table 4:** Conversion to AD in the different MCI biomarker-based subgroups

	Using Aβ42, t-Tau and *p*-Tau for classification	Using Aβ42/40 ratio, t-Tau and *p*-Tau for classification
LL	4/40 (10%)	3/38 (8%)
IAP	7/16 (44%)	8/18 (44%)
SNAP	20/36 (56%)	9/17 (53%)
HL	39/52 (75%)	50/71 (72%)

Data are expressed as n/total (percentage) of patients

*AD* Alzheimer’s disease, *MCI* mild cognitive impairment, *Aβ42* 42-aminoacid isoform of amyloid beta, *t-Tau* total Tau protein, *p-Tau* hyperphosphorylated Tau protein, *Aβ40* 40-aminoacid isoform of amyloid beta, *LL* low-AD likelihood, *IAP* isolated amyloid pathology, *SNAP* suspected non-Alzheimer pathology, *HL* High-AD likelihood

### Predictors for AD-type dementia at follow-up

Logistic regression models were employed to identify the best predictors of conversion to AD. In the first model we included age, gender, education, follow-up time, MMSE, APOE genotype and CSF Aβ42, t-Tau and p-Tau values as variables in the equation and verified that the variables that were contributing significantly to the model classification were age (*p* = 0.012), CSF Aβ42 (*p* < 0.001) and t-Tau (*p* = 0.033). We then substituted Aβ42 by the Aβ42/40 ratio in the model, and the variables retained in the model were again age (*p* = 0.015), CSF Aβ42/40 ratio (*p* < 0.001) and t-tau (*p* = 0.043). This last model that included the Aβ42/40 ratio showed a slightly better fit that the one without it, as the 2 log-likelihood or deviance (a measure for unexplained variance) was lower (91.5 *vs* 95.7, respectively). We then compared the ROC curves of the predicted probabilities derived from the two logistic regression models (Additional file 1: Figure S1). No statistically significant difference was seen between the AUC of the model including the Aβ42/40 ratio (AUC = 0.898, 95% CI 0.839–0.956) or Aβ42 (AUC = 0.879, 95% CI 0.815–0.943) (*p* = 0.181).

### Survival analysis of MCI biomarker-based subgroups

**Fig. 2:**
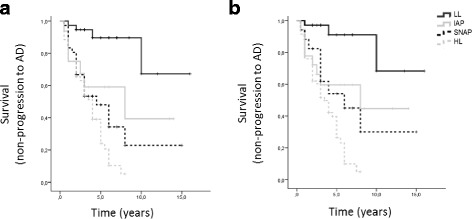
Kaplan–Meier survival curves for probability of conversion to AD according to different MCI biomarker-based subgroups. Number of individuals at risk at each time interval shown below the graphs. MCI subgroups determined taking into account CSF t-Tau and p-Tau levels and either Aβ42 (**a**) or the Aβ42/40 ratio (**b**). Log-rank (Mantel–Cox) *p* < 0.001 for both. AD Alzheimer’s disease, LL low-AD likelihood, HL high-AD likelihood, IAP isolated amyloid pathology, SNAP suspected non-Alzheimer pathology

Figure 2 shows Kaplan–Meier survival curves for the probability of conversion to AD in the different MCI biomarker-based subgroups, determined using either Aβ42 or the Aβ42/40 ratio, in combination with Tau and p-Tau. When Aβ42 was used (Fig. 2a), the LL group was significantly associated with a longer estimated time of conversion to AD (13.3 ± 1.4 years, 95% CI 10.6–15.9) than all other groups (HL, 3.6 ± 0.3 years, 95% CI 3.0–4.3, *p* < 0.001; SNAP, 6.3 ± 1.1 years, 95% CI 4.0–8.5, *p* < 0.001; IAP, 7.7 ± 1.7 years, 95% CI 4.5–11.0, *p* = 0.009). Estimated time to conversion was no different between the IAP and SNAP groups (*p* = 0.483), while a statistically significant difference was seen between the HL and IAP groups (*p* = 0.046), but not between HL and SNAP (*p* = 0.095). In Cox regression models with age, gender, education, ApoE genotype and baseline MMSE score taken into account, the difference between the HL and IAP groups was no longer significant, and a quite similar increased risk of conversion was seen in the HL (hazard ratio 7.0, 95% CI 1.9–25.6, *p* = 0.003), SNAP (hazard ratio 5.9, 95% CI 1.6–20.9, *p* = 0.006) and IAP (hazard ratio 6.3, 95% CI 1.6–25.6, *p* = 0.009) subtypes compared with patients classified into the LL group (reference). When, instead of Aβ42, the Aβ42/40 ratio was considered (Fig. 2b), a statistically decreased estimated time of conversion was again seen in HL (3.7 ± 0.3 years, 95% CI 3.1–4.2, *p* < 0.001), IAP (8.1 ± 1.5 years, 95% CI 5.1–11.1, *p* = 0.006) and SNAP (7.3 ± 1.6 years, 95% CI 4.3–2.4, *p* = 0.002) in comparison with the LL subgroup (13.5 ± 1.4 years, 95% CI 10.8–16.2). No difference was seen between the two conflicting subgroups (IAP and SNAP, *p* = 0.787), but estimated time to conversion was statistically lower in the HL group in relation both to IAP (*p* = 0.016) and SNAP (*p* = 0.029). The Cox regression model also showed that MCI patients belonging to the HL subtype had the highest risk of progression to AD (hazard ratio 10.1, 95% CI 2.2–43.0, *p* = 0.003), compared with patients classified into the LL group (reference). MCI patients classified into the IAP and SNAP subtypes also presented an increased risk of progression to AD in comparison with the LL subtype (IAP, hazard ratio 8.0, 95% CI 1.7–41.0, *p* = 0.008; SNAP, hazard ratio 6.1, 95% CI 1.2–30.7, *p* = 0.029). However, risk of progression to AD failed to reach statistical significance between the HL group and the IAP or the SNAP group. Importantly, the hazard ratio of the HL group in this model with the Aβ42/40 ratio was considerably larger than that of the same group in the Cox regression model with Aβ42.

## Discussion

In this study we investigated the effect of using the CSF Aβ42/40 ratio, instead of Aβ42 levels, both combined with Tau and p-Tau, in MCI classification according to the NIA-AA criteria. Our main findings were that the percentage of patients with amyloidosis (HL + IAP) significantly increased from 42 to 59%, reaching a similar percentage to the patients with injury markers (HL + SNAP, 56%). The proportion of interpretable biological profiles also increased significantly from 61% to 75%, particularly due to a reduction to half in the number of SNAP cases.

In our work we found an overall increase in CSF Aβ40 in MCI patients in relation to AD-dementia patients, and no difference in relation to controls. A few authors have reported on the levels of CSF Aβ40 in MCI patients, with increased [71], decreased [11] or unchanged levels [31, 32, 72] in relation to controls being reported. These discrepancies might be explained in part by technical differences in relation to the antibodies used in the different assays. While initial assays used N-terminally unspecific antibodies that could capture also N-terminally shortened Aβ40 isoforms, this has changed, and Aβ40 assays like the one we used in this work are now based on N-terminally specific antibodies. In spite of this discrepancy related to CSF Aβ40 levels, most data, including ours, point toward a decreased CSF Aβ42/40 ratio in MCI patients in relation to controls. Reasonable agreement has also been reported regarding the cutoff value for the CSF Aβ42/40 ratio, ranging between 0.05 and 0.082 [11, 23, 27–29]. In this work, we established a cutoff value of 0.068 for distinguishing AD-dementia from controls, which is in line with the results of other groups regardless of using the same commercial assay or not. In fact, in the multicenter study by Dumurgier et al. [29] the mean Aβ42/40 ratio was comparable across centers, despite the significant inter-center differences in reported CSF Aβ40 and Aβ42 levels. Therefore, this ratio seems to be less sensitive to preanalytical and analytical sources of variability both intra-laboratories [73–75] and inter-laboratories [29].

One interesting finding of our study was that CSF Aβ40 values were significantly more dispersed in MCI patients with an ambiguous profile of core CSF biomarkers. In fact, CSF Aβ40 was significantly higher in the subgroup of patients with high t-Tau/p-Tau but normal Aβ42 (SNAP), while it was lower in patients with low Aβ42 but normal levels of t-Tau/p-Tau (IAP). A similar finding had also been reported by Sauvée et al. [27] in a mixed population of patients with dementia. This supports the idea that a large inter-individual variability in Aβ load can occur and that the normalization of CSF Aβ42 concentrations to Aβ40, rather than using absolute values of Aβ42, is a more accurate measure of amyloidosis. It would be interesting to see whether indeed, as reported recently by other groups [30, 76], the Aβ42/40 ratio correlated better with amyloid load data from PET imaging than Aβ42 alone. Unfortunately, amyloid-PET was not widely available for our MCI cohort. One other possible explanation for the large dispersion in CSF Aβ40 levels that should be considered is the preanalytical variability between samples. This is considered an important confounding source for core CSF biomarkers, and can also have an impact on Aβ40 measurements, as described by others [77–79].

The use of the CSF Aβ42/40 ratio, instead of Aβ42, had a significant effect in MCI patient biomarker-based categorization. Only minor changes were seen in the classification of patients who already had a concordant biomarker profile (all markers normal or all markers abnormal). On the contrary, use of the Aβ42/40 ratio changed the classification of 50 out of 76 patients with previous ambiguous results (65.8%), and who now fell into conclusive categories. This resulted in a significant increase in the proportion of interpretable biological profiles from 61% to 75%. This is in accordance with previous results reporting that the added value of the Aβ42/40 ratio was particularly seen in patients with a discrepancy between CSF p-Tau and Aβ42, leading to a new and more informative biological conclusion [27, 29]. Overall, the most obvious effect of the use of the Aβ42/40 ratio in MCI patient classification was an increase in the percentage of patients classified into the HL group (from 31.5 to 46.5%), at the expense of a reduction in the SNAP group (from 27.9 to 12.7%). Therefore, a significant increase in the percentage of MCI patients with a positive marker for amyloidosis was also observed (from 42.1 to 58.9%), now reaching a similar percentage to the patients with injury markers. The fact that the normalization of Aβ42 using Aβ40 resulted in an approximately 50% reduction of the patients in the SNAP category is in favor of the hypothesis that a methodological bias (too conservative CSF Aβ42 cutoff values) may be underlying the unexpected high prevalence of the SNAP group reported previously [35, 37, 39]. Indeed, we observed that the mean CSF Aβ42 was lower in MCI patients who changed from SNAP to HL after taking into account the Aβ42/40 ratio than in those who remained classified as SNAP.

Amongst the 144 MCI patients who completed longitudinal clinical evaluation, no difference in baseline CSF Aβ40 was seen between MCI-St and MCI-AD patients, as already reported by others [31, 32], whereas the Aβ42/40 ratio was significantly lower in the MCI-AD group. Interestingly, our results show that both Aβ42 and the Aβ42/40 ratio were statistically different (lower) between MCI-St patients and controls, while t-Tau and p-Tau levels were comparable between the two groups. This observation might indicate that some of the subjects who have not yet progressed to AD at the time of follow-up observation, and were therefore included in the MCI-St group, will indeed progress to AD in the future, as they have decreased Aβ42 and/or Aβ42/40 ratio levels. If we consider that amyloid alterations precede neurodegeneration in AD, we may assume that these subjects are at an early stage of the disease and would progress to AD dementia if observed for a longer time. In fact, this MCI-AD/MCI-St dichotomization is completely dependent on the follow-up time, with longer periods of observation giving rise to more accurate data.

In accordance with previous studies [35, 38–40, 42], the HL group showed the highest risk of progression to AD, irrespective of the use of either Aβ42 or the Aβ42/40 ratio for biomarker-based classification. Regarding prognosis in the conflicting biomarker categories, in our study the percentage of MCI patients classified as IAP or SNAP who converted to AD was not very different from each other (close to 50%) and did not differ much whether CSF Aβ42 alone or the Aβ42/40 ratio, combined with Tau and p-Tau, was used. Nevertheless, the results depicted in Fig. 1 show that, within MCI patients who progressed to AD, the use of the Aβ42/40 ratio resulted in a significant increase in the percentage of patients classified as HL and a decrease in the percentage of patients classified as SNAP. Survival analysis showed that, when Aβ42 was used for MCI subgroup classification, both IAP and SNAP had significantly lower estimated time of conversion to AD than the LL group, that was similar to the HL group in the case of SNAP but not in the case of the IAP subgroup. The inclusion of the Aβ42/40 ratio in MCI biomarker-based subgroup classification decreased the overlap between the HL and the SNAP survival curves, with both conflicting subgroups now showing an estimated time of conversion to AD that was significantly lower than the LL subgroup and higher than the HL subgroup. In Cox regression models, the same overall effect of the Aβ42/40 ratio on the risk of progression to AD was seen. In the model that used Aβ42, risk of progression to AD was very similar between the HL, IAP and SNAP subgroups. When we used the Aβ42/40 ratio instead, the difference between the hazard ratios of the three potential risk categories (IAP, SNAP and HL) increased. Both the IAP and SNAP categories presented with an equivalently increased risk of progression to AD, in comparison with the LL subtype, that was numerically lower than the HL group, but failed to reach statistical significance. More importantly, when comparing the hazard ratio of the HL group in both Cox regression models, it is evident that this is higher in the model that includes the Aβ42/40 ratio than in the model that includes Aβ42 (10.1 vs 7.0). This is also true for the IAP category (8.0 vs 6.3), but not for SNAP (6.1 vs 5.9). This finding is indicative that use of the Aβ42/40 ratio for patient classification indeed results in a better predictive value of future conversion to AD dementia. This was not confirmed by our logistic regression models, however, that failed to reach a statistically significant difference between the AUCs when including the Aβ42/40 ratio or Aβ42 in the models. Although some of our group analysis did not prove statistically that the Aβ42/40 ratio predicted conversion to AD with more accuracy compared to Aβ42, we believe the fact that it reduced conflicting biomarker results and produced biomarker-based subgroups with more clear-cut differences in the risk of progression to AD is of value for individual MCI patient clinical follow-up.

Some limitations of the current study must be addressed. The fact that CSF Aβ40 assays (that only became available in our laboratory in 2016) were all performed within a short period of time in previously stored samples, and not in a sequentially routine setting, as the other CSF biomarkers, might introduce some methodological bias to the results, and can contribute to the ratio having a better performance than Aβ42 alone. As already mentioned, imaging biomarkers were not considered in this study, as the availability of amyloid-PET data was very scarce. It would be interesting to correlate the CSF amyloid assessment either through Aβ42 or the Aβ42/40 ratio with this alternative diagnostic tool, categorizing the population as amyloid-positive or negative. Since only the amnestic subtype of MCI was considered, the generalization of the results to other forms of MCI should be cautious. Finally, as in many other clinical studies, no neuropathological verification was available, leaving the possibility of misdiagnosis. However, this study was developed in this specific context of routine clinical practice and we believe that this is a strength of the present work. Since we enrolled patients in a systematic way, our cohort may be considered representative of an ordinary tertiary Memory Clinic, surpassing the selection biases of investigational studies. Also, the fact that our data are based on a variable length of follow-up (≥ 2 years) not only optimizes the available study information, but also reduced the chances of underestimating the predictive power of the selected parameters that might occur with a short fixed follow-up period. In addition, the rigorous methodology adopted to define stages and progression, the use of neuropsychological instruments well-validated for the Portuguese population and administered by the same experienced team of neuropsychologists, as well as the standardized use of the CSF biomarkers may also improve the reliability of the results.

## Conclusion

Our results confirm the usefulness of the addition of the CSF Aβ42/40 ratio in the interpretation of the CSF profile of MCI patients, as it increases the proportion of patients with conclusive profiles, therefore enhancing their predictive value for underlying AD dementia.

## Additional files

Additional file 1: **Figure S1.** Receiver operating characteristics (ROC) curves for (A) distinguishing between AD-dementia patients and controls and for (B) the predicted probabilities of conversion to AD in MCI patients derived from logistic regression models, using either CSF Aβ42 or the Aβ42/40 ratio. (PDF 59 kb)

Additional file 2: **Table S1.** Demographic, clinical and genetic data of the MCI subgroups based on a CSF biomarker profile that included the Aβ42/40 ratio, t-Tau and p-Ta. (PDF 216 kb)

## Abbreviations

AD: Alzheimer’s disease
ADAS-Cog: Alzheimer Disease Assessment Scale—Cognitive
ANOVA: Analysis of variance
APOE: Apolipoprotein E
APP: Amyloid precursor protein
AUC: Area under the receiver operating characteristics curve
Aβ: Amyloid beta
Aβ40: 40-aminoacid isoform of amyloid beta
Aβ42: 42-aminoacid isoform of amyloid beta
BLAD: *Bateria de Lisboa para Avaliação de Demência* (Lisbon Battery for Dementia Assessment)
CDR: Clinical Dementia Rating scale
CI: Confidence interval
CNS: Central nervous system
CSF: Cerebrospinal fluid
CT: Computed tomography
CV: Coefficient of variance
DAD: Disability Assessment for Dementia
DSM-IV-TR: *Diagnostic and Statistics Manual for Mental Disorders*—fourth edition
EDTA: Ethylenediamine tetraacetic acid
ELISA: Enzyme-linked immunosorbent assay
HL: High-AD likelihood
IAP: Isolated amyloid pathology
LL: Low-AD likelihood
MCI: Mild cognitive impairment
MCI-AD: Mild cognitive impairment patients who progress to Alzheimer’s disease
MCI-St: Stable mild cognitive impairment
MMSE: Mini Mental State Examination
MoCA: Montreal Cognitive Assessment
MRI: Magnetic resonance imaging
NIA-AA: National Institute of Aging–Alzheimer Association
NINCDS-ADRDA: National Institute of Neurological and Communicative Disorders and Stroke–Alzheimer’s Disease and Related Disorders
NPI: Neuropsychiatric Inventory
PCR-RFLP: Polymerase chain reaction-restriction fragment length polymorphism
PET: Positron emission tomography
p-Tau: hyperphosphorylated Tau protein
ROC: Receiver operating characteristics
SD: Standard deviation
SNAP: Suspected non-Alzheimer pathology
SPECT: Single-photon emission computed tomography
SPSS: Statistical Package for the Social Sciences
STARD: Standards for Reporting of Diagnostic Accuracy
t-Tau: total Tau protein
