# Supplementary material for: Addition of the Aβ42/40 ratio to the cerebrospinal fluid biomarker profile increases the predictive value for underlying Alzheimer’s disease dementia in mild cognitive impairment
Source: Alzheimers Res Ther. 2018 Mar 20;10:33. doi: 10.1186/s13195-018-0362-2 (PMC5861634; doi:10.1186/s13195-018-0362-2)
Supplement: Supplementary file 1 — Figure S1. Receiver operating characteristics (ROC) curves for (A) distinguishing between AD-dementia patients and controls and for (B) the predicted probabilities of conversion to AD in MCI patients derived from logistic regression models, using either CSF Aβ42 or the Aβ42/40 ratio. (PDF 59 kb) [file 13195_2018_362_MOESM1_ESM.pdf]

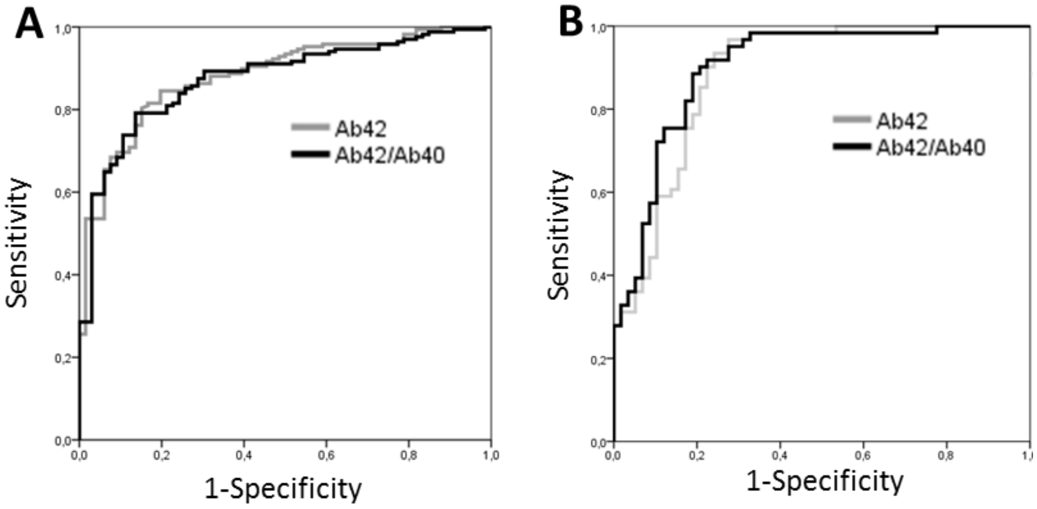

Figure S1: Receiver operating characteristics (ROC) curves for (A) distinguishing between AD-dementia patients and controls and for (B) the predicted probabilities of conversion to AD in MCI patients derived from logistic regression models, using either CSF Aβ42 or the Aβ42/40 ratio.
