# Supplementary material for: Addition of the Aβ42/40 ratio to the cerebrospinal fluid biomarker profile increases the predictive value for underlying Alzheimer’s disease dementia in mild cognitive impairment
Source: Alzheimers Res Ther. 2018 Mar 20;10:33. doi: 10.1186/s13195-018-0362-2 (PMC5861634; doi:10.1186/s13195-018-0362-2)
Supplement: Supplementary file 2 — Table S1. Demographic, clinical and genetic data of the MCI subgroups based on a CSF biomarker profile that included the Aβ42/40 ratio, t-Tau and p-Ta. (PDF 216 kb) [file 13195_2018_362_MOESM2_ESM.pdf]

Table S1 – Demographic, clinical and genetic data of the MCI subgroups based on a CSF biomarker profile that included the Aß42/40 ratio, t-Tau and p-Ta.

	Low-AD likelihood	High-AD likelihood	IAP	SNAP
N (%)	56 (28.4%)	92 (46.7%)	24 (12.2%)	25 (12.7%)
Gender (M/F)	18/38	33/59	7/17	10/15
Age (years)	61.3±9.9	69.5±8.2***	69.2±7.6**	69.3±8.7**
Age onset (years)	58.1±10.1	67.0±8.3***	63.9±8.3	66.2±6.1
Education (years)	6.9±3.8	5.8±3.9	4.5±2.2	6.8±4.9
MMSE	27.6±2.5	24.8±4.1***	25.5±5.8	26.7±2.8
MoCA	21.0±4.5	16.5±5.4***	15.8±6.9**	17.8±4.4
ADAS-Cog	8.4±4.6	13.8±6.1***	12.8±7.1	10.6±4.9
ApoE-ε4 (%)	22.2%	51.2%***	33.3%γ	56.5%
Aβ42 (pg/mL)	890±285	539±213***	533±230***,§§§	928±418γγγ
Aβ40 (pg/mL)	8357±3040	13112±5095***	11126±2902**	10234±3738
Aβ42/40	0.113±0.037	0.042±0.014***	0.048±0.015***,§§§	0.089±0.018γγγ
t-Tau (pg/mL)	161±47	549±276***	180±53γγγ,§§§	407±176***
p-Tau (pg/mL)	29±8	69±29***	31±10γγγ,§§§	49±14***
Follow-up time (years)	3.9±3.4	3.7±2.3	4.5±3.8	4.4±3.4

Data are expressed as mean ± S.D, except for *APOE* that is expressed as percentage of ε4 carries. M – male; F – female; IAP – Isolated Amyloid Pathology; SNAP – Suspected Non-Alzheimer Pathology. MMSE and MoCA: higher scores correspond to better performance; ADAS-Cog: lower scores correspond to better performance. *P<0.05 vs. low-AD-likelihood. **P<0.005 vs. low-AD-likelihood. ***P<0.001 vs. low-AD-likelihood. γP<0.05 vs. high-AD-likelihood. γγγ P<0.001 vs. high-AD-likelihood. §§§P<0.001 vs. SNAP.
